# Applying the consolidated framework for implementation research to evaluate the community rapid intervention service

**DOI:** 10.1186/s12913-023-09864-z

**Published:** 2023-08-09

**Authors:** Alice Moult, Dereth Baker, Helen Twohig, Matthew Missen, Zafar Iqbal, Helen Duffy, Zoe Paskins

**Affiliations:** 1 School of Medicine, Keele University, Newcastle-Under-Lyme, ST5 5BG UK; 2 Midlands Partnership University NHS Foundation Trust, Trust Headquarters, Corporation St, Stafford, UK; 3 Haywood Academic Rheumatology Centre, Haywood Hospital, Midland Partnership University NHS Foundation Trust , ST5 5BG Stafford, UK

**Keywords:** Evaluation, Health services, Qualitative, Implementation

## Abstract

**Background:**

Developed in 2019, the Community Rapid Intervention Service (CRIS) is a community intervention service aiming to prevent hospital admissions. CRIS provides a response within two hours to patients with sub-acute medical needs in their usual place of residence. This evaluation aimed to identify challenges and facilitators to implementation of the service, with a view to informing future service development, optimising patient care and disseminating learning to other areas looking to implement similar services.

**Methods:**

This study used the Consolidated Framework for Implementation Research (CFIR) as an evaluation framework. We conducted semi-structured interviews with local healthcare system leaders, clinicians that worked within the CRIS, and clinicians who interfaced with the CRIS. The CFIR was used to guide data collection and analysis. Two Community of Practice (CoP) meetings were held to gather stakeholders’ perspectives of the evaluation.

**Results:**

Three key themes were identified from the analysis of 13 interviews: contextual factors influencing implementation, service identity and navigating complexity. Contextual factors such the influence of the Covid 19 pandemic upon health services and the expansion of the CRIS were discussed by participants. The adaptability of the service was deemed both a facilitator and challenge of implementation. Ways to build-on and improve the existing CRIS model were suggested.

**Conclusion:**

This evaluation has shown that the CRIS may need to be redefined with clarity provided as to how the service interfaces with other urgent and planned care offered in acute, primary, community and social services. Structuring the evaluation around the CFIR was helpful in identifying facilitators and challenges that influenced the implementation of the CRIS.

**Supplementary Information:**

The online version contains supplementary material available at 10.1186/s12913-023-09864-z.

## Background

Within the United Kingdom (UK) models of urgent care in the community are delivered across multiple services (e.g. Urgent Care Response and End of Life services) [1]. Models of urgent care in the community are commonly characterised by multiple points of access and differentiated acceptance and exclusion criteria that makes referral into these services complex [2]. Unlike previous models, the Community Rapid Intervention Service (CRIS) has a single point of access and is an integrated cross-organisational service delivered by staff from one Acute Care Trust and one large Community Services Trust. A single point of access may be beneficial as it could reduce the number of inappropriate referrals into the service [3].

## The community rapid intervention service

The CRIS was developed in North Staffordshire and Stoke-on-Trent in September 2019. The service provides a rapid two hour response to patients with sub-acute medical needs in their usual place of residence offering clinical assessment and consultant physician input into community care and management within a virtual ward for up to five days [3]. Sub-acute medical conditions may include, but are not limited to: chest infections, urinary infections, falls, delirium, Chronic Obstructive Pulmonary Disease (COPD) exacerbations, cellulitis, medication-related problems and wound infections. The CRIS team consists of call handlers, clinical triage nurses, Advanced Nurse Practitioners (ANPs) and hospital consultants working in partnership. After initial triage, an ANP will assess the patient and then discuss further investigations and management with consultants.

Referral into the CRIS comes from an Unscheduled Care Coordination Centre (UCCC) based in North Staffordshire [4]. The UCCC provides a single access point which offers clinical advice, support and referral onto the most appropriate service. General Practitioners (GPs), care homes, paramedics and NHS 111 can all contact the UCCC.

The aim of CRIS is to reduce Accident and Emergency (A & E) attendance and admission to hospital medical wards which, in turn, has the potential to deliver benefit to the local healthcare system through efficiencies in patient care [3]. The CRIS also supports system efforts to improve bed occupancy by reducing the length of stay for non-elective patients admitted to medical wards and by supporting Medically Fit for Discharge (MFFD) planning through the Oximetry at home [3]. At the time of this evaluation the CRIS had recently expanded into South Staffordshire, UK.

To inform future service development, and to optimise patient care, a need arose to evaluate the implementation of the CRIS [5, 6]. When the CRIS was originally developed it was a novel community care service. Other regions are looking to develop similar models; evaluating the CRIS could inform both the development and implementation of such models to meet with national NHS England standard of two hour urgent community response [7]. This evaluation aimed to identify facilitators and challenges to the implementation of the service. Identifying these factors will, in turn, optimise service delivery and system efforts to provide effective models of care in the community. An independent qualitative evaluation provides an ability to gain an in-depth understanding of the CRIS from multiple stakeholder perspectives.

## Methods

This qualitative evaluation was one part of a larger evaluation which had quantitative and qualitative data collection.

### The framework

The qualitative evaluation was guided by the Consolidation Framework for Implementation Research (CFIR) which supports rapid-cycle evaluation of the implementation of healthcare services, and produces actionable findings intended to improve implementation in a timely manner [8–10]. The CFIR is a meta-theoretical framework particularly well-suited to this evaluation given its ability to identify key barriers and facilitators of implementation from the perspective of multiple stakeholders. The CFIR has 39 constructs organised into five major domains: i) intervention characteristics (eight constructs), ii) outer setting (four constructs), iii) inner setting (14 constructs), iv) characteristics of individuals (five constructs) and, v) process (eight constructs) [9]. Table 1 lists all the CFIR domains and constructs. Since the completion of data collection in this evaluation the CFIR has been updated [10].

**Table 1:** CFIR domains and constructs

Domain	Constructs
Intervention characteristics	• Intervention source • Evidence strength and quality • Relative advantage • Adaptability • Trialability • Complexity • Design quality and packaging • Cost
Outer setting	• Patient needs and resources • Cosmopolitanism • Peer pressure • External policy and incentives
Inner setting	• Structural characteristics • Networks and communication • Culture • Implementation climate • Readiness for implementation
Characteristics of individuals	• Knowledge and beliefs about the intervention • Self-efficacy • Individual stage of change • Other personal attributes
Process	• Planning • Engaging • Executing • Reflecting and Evaluation

The aim of this study was to use the CFIR as an evaluation framework to identify challenges and facilitators to implementation that need to be addressed to optimise the delivery of the CRIS.

### The study design

A qualitative approach, informed by the Framework method [9, 10] was used. Semi-structured interviews were conducted with:

- local healthcare system leaders,
- clinicians working within the service,
- clinicians who interface with the service including: GPs, professionals in adult social care, palliative care and individuals who work for West Midlands Ambulance Service (WMAS).

The analysis was led by a Research Follow in Knowledge Mobilisation (AM). The analytic team also included a Research Assistant with substantive experience of working non-clinically within the NHS (DB) and an academic GP (HT).

### Community of practice

To guide the qualitative evaluation a Community of Practice (CoP) was convened by researchers at Keele University and commissioners of this evaluation. A CoP is a group of people who share a concern or a passion for a phenomena and learn as they interact with each other [11]. The CoP was facilitated by staff members within the Impact Accelerator Unit (IAU) based within Keele Medical School. The aim of developing a CoP was twofold:

- to facilitate communication and dialogue between different stakeholder groups involved in, or using the CRIS.
- to use stakeholders’ views to guide the evaluation and implementation plan.

Two CoP meetings were held. The research team envisaged holding more than two meetings but were limited by the time constraints of the project and significant pressures within the NHS to deliver services. The first CoP meeting was held on the 6th May 2022. The aim of the first session was for stakeholders of the CRIS to:

- understand the CRIS and how the service operates.
- to discuss the qualitative evaluation of the CRIS.

Commissioners of the evaluation sent out email invitations to individuals, who they perceived to be stakeholders of the service, inviting them to the CoP. A total of 26 people from a variety of academic and clinical backgrounds attended. Three attendees were public contributors. When discussing the CRIS, attendees perceived that the service was beneficial but an evaluation was needed to improve service delivery due to the lack of clarity of service offerings.

A second CoP was held on the 10th October 2022. The aim of the second session was to:

- discuss the findings of the service evaluation.
- review and refine interpretation of the interviews.

Similar to the first CoP, commissioners of the evaluation sent out email invitations to individuals, who they perceived to be stakeholders of the service, inviting them to the CoP meeting. A total of 15 people from a variety of academic and clinical backgrounds attended along with two public contributors. The discussion of the findings informed recommendations on how to improve service delivery.

### Sampling of participants for interviews

Interview participants were purposefully sampled from the first Community of Practice (CoP) meeting. Local healthcare system leaders, clinicians working within the service and clinicians who interface with the service were invited to participate in an interview.

The criteria for inclusion were any leader or healthcare professional that works within the CRIS, or any healthcare professional that interfaces with the service. Leaders of the CRIS include professionals who are in the position to guide or direct the service within North and/or South Staffordshire. Leaders or healthcare professionals were excluded if they did not work within, or interface with, the CRIS.

### Data collection

The evaluation of the CRIS took place between April 2022 and October 2022. All interviews were conducted by AM via Microsoft Teams and were digitally recorded and transcribed. Only AM and the participant were present during the interview. Interviews lasted from 20 to 65 min. Recruitment ceased once data saturation, defined as when no new data was being coded within the framework [12], was reached.

The questions asked within the interviews were informed by the CFIR. Topic guides (outlines of key issues and areas of questioning used to guide a qualitative interview) [13] were therefore designed to encompass key constructs of the five CFIR domains. The research team iteratively developed separate but related topic guides based on participant role: local healthcare system leader, clinician who worked within the service or clinician who interfaced with the service.

AM had no pre-established relationship with any of the participants. The interviewer being external to the CRIS was important as it provided participants with the opportunity to provide complete and honest responses.

Consent and demographic information (e.g. age, gender, and clinical background) were electronically obtained from all participants prior to the interview commencing.

### Data analysis

The process of analysis followed a modified version of the framework method [14, 15]. The first step of framework analysis – the transcription of data – was conducted by a transcription company external to the study team. The procedure for analysis included: data familiarisation (step one), developing an analytical framework (step two), applying the analytical framework (step three), charting data into a framework matrix (step four) and interpreting the data (step five).

Research analysts (AM, DB or HT) read each interview transcript to immerse themselves within the data (step one). This familiarisation process was essential in cases where the researcher analysing the data had not been present during the interview. The CFIR was used as an analysis framework to structure and organise the data. Initially the research analysts independently coded data line by line from the same three interview transcripts onto a Template Summary Table comprised of the CFIR domains (please see Additional File 1 for an example). Each data extract could be coded against more than one of the CFIR domains, if deemed appropriate. If the data did not fit into any of the CFIR domains a new code was generated which reflected the language and experiences of participants (step two).

After the research analysts coded the same three transcripts they met and discussed any discrepancies until a consensus was reached. Generally, researchers coded the same data to CFIR domains. Two additional codes were discussed and integrated into the framework which were virtual wards and geographical location (step three). The research analysts then applied the final analytical framework to each interview transcript (step four). The data was then summarised into a framework matrix (step five). The matrix comprised of one row per CFIR domain. Researchers then abstracted data from each participant’s Template Summary Table and inserted it into the corresponding cell within the matrix (please see Additional File 2 for an example).

During a series of meetings, analysts collaboratively and iteratively reviewed the matrix and discussed the CFIR domains. When reviewing the matrix analysts noted that the data mapped onto the domains and constructs sometimes did not strictly fit within the definition provided by the CFIR framework, nor did it fit into any other domains. Some findings were also duplicated across domains. The data mapped onto the ‘Complexity’ construct described a confusion about service offerings due to unclear inclusion and exclusion criteria, a key challenge of implementing the service. Yet, the definition provided by the CFIR framework defines ‘Complexity’ as the perceived difficulty of implementation. Furthermore, some of the data mapped to ‘Adaptability’ related to the need to clearly and deliberately define the service (another key challenge to implementation), but did not explicitly describe the degree to which an intervention can be adapted, tailored refined, or reinvented as suggested by the CFIR framework. Analysts also noted that the data mapped to ‘Cosmopolitanism’ did discuss how the service was networked with other services, which fits within the CFIR definition, but also included ways to improve relationships between the networks (e.g. through education).

The influence of the Covid 19 pandemic upon healthcare services was mapped to various domains and constructs (e.g. Adaptability and Complexity). Analysts thought that there was no domain or construct that explicitly captured external influences such as the Covid 19 pandemic, which was another challenge to the implementation of the CRIS.

Through discussions with the wider research team, analysts re-framed the data into themes to better reflect participants’ descriptions of challenges and facilitators of implementation. Themes were generated from the data set by reviewing the matrix and making connections within and between participants and domains. Initial findings were shared at the second CoP meeting to help interpretation and to generate meaning from the data.

Analysis was initiated when the first interview was completed and continued concurrently with data collection to help determine when data saturation [12] had been reached.

### Ethics approval

The qualitative interviews received ethical approval from the NHS Research Ethics Committee (REC; reference: 22/SW/0075).

### Service users and carers recruitment

To recruit service users and carers, clinicians who worked within the CRIS were asked by the research team to distribute advertisements directly to service users and carers when they came into contact with them. The research team received no expressions of interest to participate in the study from service users and carers. Due to time constraints, the study team decided it was not possible to explore other recruitment avenues.

## Results

The sample consisted of five local healthcare system leaders, one clinician working within the CRIS and seven clinicians interfacing with the service. There were 7 males and 5 females with an age range between 42 and 61 years. Participants’ experience of working within their current role ranged between 12 months and 32 years.

Three themes were identified that reflected participants’ descriptions and experiences with the CRIS: 1) Contextual factors influencing implementation, 2) Service identity and 3) Navigating complexity. Table 2 shows the relationships between the themes, CFIR domains and constructs and the key implementation challenges and facilitators relating to each theme. The highlighted rows indicate where the data mapped onto the CFIR domains and constructs did not strictly fit within the CFIR definitions of the domains and constructs.

**Table 2:**
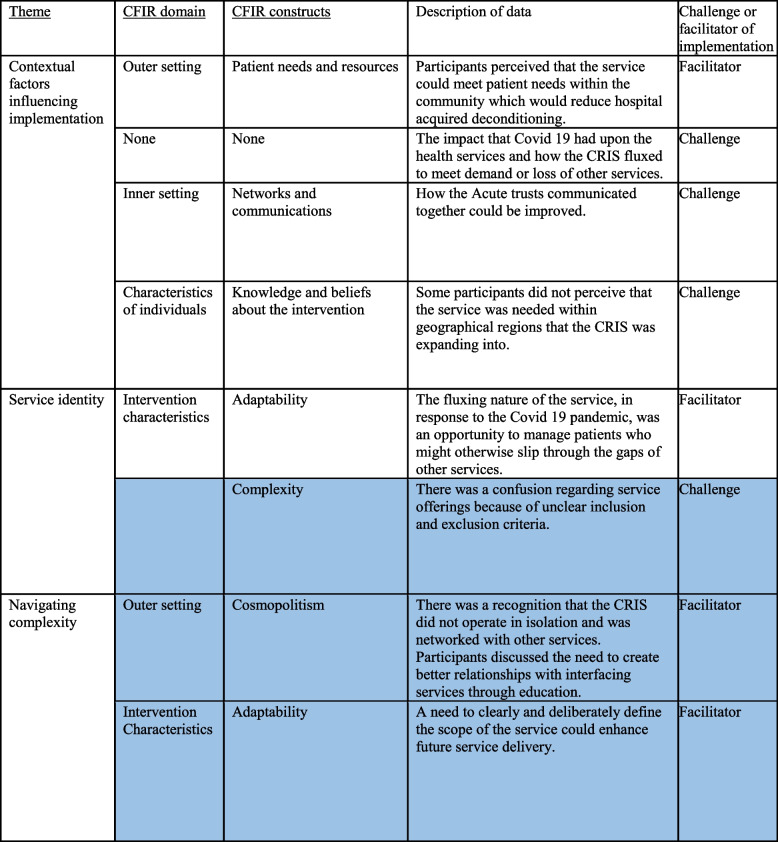
Themes, CFIR domains and constructs

The highlighted rows indicate where the data mapped onto the CFIR domains and constructs did not strictly fit within the CFIR definitions of the domains and constructs

### Contextual factors influencing implementation

Contextual factors such as needs of the local patient population, the influence of the Covid 19 pandemic upon health services, the expansion of the CRIS into another geographical region and the partnership between the Acute Care Trust and Community Services Trust were all discussed by participants.

The main facilitator of implementation was perceived patient benefit. In the face of constant pressures on A & E, all participants suggested that the CRIS provided patients with the opportunity to have their needs met within the community without hospital acquired deconditioning or risk of infection. Whilst participants recognised the benefits of the service, a number of participants reflected on how the service had been developed prior to the Covid 19 pandemic and how the healthcare system had changed. Since the pandemic, and the subsequent pressure put on National Health Service (NHS), there has been a loss of services. Participants reflected on how the CRIS was trying to “*fill in”* the gaps left by the loss of other services. Building upon this, a few participants also suggested that some patients were inappropriately referred into the service by the Unscheduled Care Coordination Centre (UCCC) due to pressure or loss of other services:

*“If I contacted the Unscheduled Care Co-ordination Centre I’m not necessarily after CRIS, there might be other services that may be relevant but because of the loss of those services CRIS would end up picking up slack.”* Participant Eight

Whilst the CRIS was trying to meet demands on healthcare services, it was also expanding into another geographical location (South Staffordshire). The expansion of the CRIS was discussed by all participants with most describing different healthcare contexts between North and South Staffordshire. All participants suggested that the service was originally developed to reduce unprecedented A & E attendances within an Acute Care Trust based in North Staffordshire. Yet, most participants perceived that there was not the same volume of people presenting to A & E within South Staffordshire thus reducing the need for the service. One participant stated *“I was never convinced that South Staffordshire needed the same service anyway.”* Furthermore, some participants suggested that there was already existing admission avoidance work within South Staffordshire and they were concerned that the implementation of the CRIS would influence established relationships with interfacing services:

*“Well how are we gonna stop it duplicating what we’ve already got? How are we going to move you know – there’s the staff that are already doing this work and we’ve got really good relationships with the GPs, aren’t we just going to breakdown a lot of good stuff that we’ve got to create this CRIS that we don’t really need?”* Participant Nine

The perceived implementation driver seemed to be equity of service provision across the areas, not need for the service. Some participants reported that a lack of perceived need for the service within South Staffordshire may inhibit the involvement of key stakeholders.

Another important contextual factor which influenced the implementation of the CRIS was the relationship between the Acute Care Trust and Community Services Trust. A majority of participants described the importance of both Trusts working in partnership, listening and taking on board each other’s views to provide integrated care. When discussing the process of implementation, participants recognised the hard-working ethos of staff working within the Acute Care Trust, one participant suggested: *“They are do-ers. Bang, bang, bang and it’s done.”* Whilst staff working within the Acute Care Trust were deemed operationally strong, some participants suggested that they had taken ownership of the service and sometimes did not communicate changes in service provision to staff working within the Community Services Trust. Whilst the perceived lack of engagement was seen to be a challenge when implementing the CRIS, a number of participants discussed that there was an opportunity to further develop the partnership between the Trusts and to collaborate and share learning to improve the CRIS model of working together.

#### Service identity

Most participants described that the CRIS had adapted since it’s pre-pandemic initiation:

*“I think during COVID they then ended up getting involved in some other stuff with care homes which muddied the water even more. They then got involved with – I think they got involved with the long-winded COVID pathway, they got involved with – I can’t remember how many things that CRIS was the answer to during that time. So it lost its way. It seems very reactive though.”* Participant Nine

The adaptability of the service was deemed both a facilitator and challenge of implementing the CRIS. The evolving and responsive nature of the CRIS meant that it became a service which was much broader and complex in nature than first anticipated. One participant suggested that the CRIS had become a “*one-stop shop for everybody*.” Yet, a few of the local healthcare system leaders viewed the adaptability of the service in a positive light describing that this allowed the CRIS to manage patients who otherwise might “*slip through the gaps”* of other interfacing services. Other participants suggested that the adaptability of the CRIS created confusion about the role of the service with one participant stating:

*“What problem is the CRIS service trying to fix? And if the CRIS service is going to fix everybody’s problems then is just needs to stay as it is and just become a bigger and bigger monster.”* Participant Nine

The confusion about service offerings limited the effective use of the CRIS. One participant, who interfaced with the service, stated: “*I don’t really understand what the CRIS team actually do.*” Other participants who interfaced with the service provided examples of instances when they perceived that the CRIS was not accepting patients who could be treated within the community. One GP participant gave the following example:

*“I just wanted CRIS team to over the weekend you know, to have a call or see the patient over the weekend and then, I was happy with that and they would not accept that. You know, they said well, you need to send this patient to A&E because they were not happy to take them.”* Participant Seven

A lack of clarity regarding acceptance and exclusion criteria prevented participants who interfaced with the service from utilising the CRIS. This lack of clarity was exacerbated by these participants’ misunderstanding of the structural characteristics of the service, particularly referral pathways. Participants who interfaced with the service described directly contacting the CRIS without recognising the role of the UCCC.

#### Navigating complexity

The CRIS sits within a wider network of services meaning that there is a high degree of cosmopolitanism (the degree to which an organisation is networked with other external organisations [8]) which makes the implementation of the service complex. All participants suggested that the CRIS’ provision needed to be set out in the context of the services with which it interfaces (e.g., primary, community and social care) and that they all need to work cohesively together to manage patients. To do this, and to further facilitate the implementation of CRIS, participants described that existing relationships with interfacing services could be strengthened. A number of participants suggested more involvement from stakeholders who work within interfacing services, especially primary care. Throughout the process of implementing the CRIS these individuals could be ‘champions’ of the service.

To ensure all stakeholders know the remit of the CRIS, a local healthcare system leader suggested engaging and educating interfacing services on what the CRIS offers:

“*I think probably better education with some of the [interfacing services] as well about what the [interfacing service’s] expectation is*.” Participant Three

A key feature within the education of interfacing services could be the structural characteristics of the CRIS, and that the UCCC was the single point of access:

*“…And I’d want to make sure that the Unscheduled Care Coordination Centre was known as the single point of access [and] all those pathways were as clear as anything.”* (Participant Five)

The overall data-set suggested that the CRIS is at a critical point in the service’s development. Most participants suggested that the CRIS could not continue to be as responsive to healthcare needs as it could “*not fill all gaps*” within the healthcare service. A majority of participants suggested that, due to the adaptability of the service, local healthcare system leaders needed to clearly define the scope of the CRIS to enhance future service delivery.

## Discussion

The aim of this evaluation was to identify challenges and facilitators to implementation of the CRIS, with a view to informing future service development, optimising patient care and disseminating learning to other areas looking to implement similar services. The main facilitator of the CRIS’ implementation was that the service was seen to meet patients’ needs within the community avoiding hospital admissions and subsequent harms associated with hospital stays. Implementation challenges included: the influence of the Covid 19 pandemic upon healthcare services, the expansion of the CRIS into South Staffordshire, the opportunity for a more collaborative partnership between the Acute Care Trust and Community Services Trust, confusion regarding service offerings and the lack of clarity regarding acceptance and exclusion criteria.

Similar to previous studies, this evaluation has shown that a complex health service adapts, interacts and co-evolves with other interfacing health services [16–18]. The NHS Priorities and Operational planning guidance published in 2022 [7] recommends that services improve the responsiveness of Urgent and Emergency Care (UEC) and build community care capacity; keeping patients safe and offering the right care, at the right time, in the right setting. In order to do this, interfacing services need to clearly know CRIS’ service offerings.

Previous literature has suggested that having a single point of access could reduce the number of inappropriate referrals into a service [3]. The findings of this evaluation have identified that despite even with a single point of access in the Unscheduled Care Coordinate Centre (UCCC), integrated with the CRIS, healthcare professionals remain unclear on CRIS’ service offerings. The UCCC as a single point of access provides a contact point but this evaluation suggests that the UCCC is referring patients into the CRIS to prevent hospital attendance or admission. This was attributed to a loss of capacity in alternative services (e.g. Primary Care), subsequent increased pressure in unplanned and emergency care services and the view that CRIS is a broader admission avoidance service beyond the original service scope. To address this, and to clarify service scope, inclusion and exclusion criteria for the CRIS should be clearly defined, taking into account the demand on other health services within the local area.

Studies within the implementation literature suggest that interventions must be tailored to fit within different contexts, and it is important for stakeholders to believe that there is a need for the service [19]. This evaluation has shown that if a health service is initiated in a new location without a perceived need (e.g. the CRIS expanding into South Staffordshire), this may inhibit the involvement of key stakeholders. Furthermore, how to establish effective integrated working when jointly running a community intervention service remains unclear within the academic literature. This evaluation has shown that there is an opportunity for healthcare partners to learn from each other by taking a more collaborative approach when working together.

Previous studies have used the CFIR to identify constructs which may, or may not, lead to implementation success [20]. In this evaluation, the CFIR provided a framework to systematically identify factors that emerged in various contexts to influence implementation. The two constructs which were particular challenges of implementation, and seemed to be connected, were Adaptability and Complexity. The adaptability of the CRIS made the service complex in nature. Participants discussed the need to define the fluxing service which was not explicitly captured throughout the CFIR domains. Additionally, similar to other implementation science frameworks [21], we found that the CFIR would be better suited to assess the implementation of clearly structured interventions; this could be because the field of implementation science has largely developed frameworks for institutional settings, rather than fluxing community settings [22]. Despite this drawback, the CFIR framework was helpful for identifying key factors which influenced implementation and identifying ways to optimise future service delivery.

Although data saturation was reached, the findings may be limited by the sample size. Whilst the number of interviews was small, participants included those who had experiences working within, leading and interfacing with the CRIS. All participants had first-hand experiences of the implementation of the CRIS. Discussions within the second CoP suggested that the findings of this qualitative evaluation were accepted as reflective of the system’s response to establishing and adapting the CRIS service. Although the evaluation intended to recruit patients none were recruited and, therefore, their perceptions of the service are missing.

## Conclusions

Participants’ positive beliefs about the service was the main facilitator of implementation. However, to ensure that the CRIS is meeting patient and carer’s needs, an evaluation of their experience and perceptions of CRIS is needed.

To successfully implement the CRIS, challenges need to be addressed which include stakeholders working with, and within, the service. Stakeholders working within the CRIS could collaboratively define the inclusion criteria and agree the strategic direction of the service as part of the Integrated Care Partnership (ICP) strategy and delivery plan for Urgent Care, and could align this strategy to the NHS Priorities and operational planning guidance [7]. Furthermore, educating interfacing services on the CRIS’ offerings would be beneficial.

This evaluation supports prior literature for using the CFIR to evaluate the implementation of complex interventions. Whilst the CFIR is more useful when applied to institutional interventions and would benefit from modifications to better capture more fluid interventions, structuring this evaluation around the CFIR was useful in identifying multiple facilitators and challenges which are important to acknowledge to optimise service delivery.

### Supplementary Information

**Additional file 1.** Example template summary table.

**Additional file 2.** Example from matrix.

## Data Availability

Data for this study will be made available to the scientific community on request after publication. Data will be made available for scientific purposes for researchers whose proposed use of the data has been approved by a publication committee. Data and documentation will be made available through a secure file exchange platform after approval of proposal and a data transfer agreement is signed (which defines obligations that the data requester must adhere to regarding privacy and data handling). Partially deidentified participant data limited to the data used for this work will be made available. For data access, please contact the corresponding author.

## Abbreviations

A & E: Accident and Emergency
ANP: Advanced Nurse Practitioner
CFIR: Consolidated Framework for Implementation Research
COP: Community of Practice
COPD: Chronic Obstructive Pulmonary Disease
CRIS: Community Rapid Intervention Service
GP: General Practitioner
IAU: Impact Accelerator Unit
NHS: National Health Service
UCCC: Unscheduled Care Coordination Centre
WMAS: West Midlands Ambulance Service
