# Supplementary material for: Applying the consolidated framework for implementation research to evaluate the community rapid intervention service
Source: BMC Health Serv Res. 2023 Aug 9;23:844. doi: 10.1186/s12913-023-09864-z (PMC10413526; doi:10.1186/s12913-023-09864-z)
Supplement: Supplementary file 1 — Additional file 1. Example template summary table. [file 12913_2023_9864_MOESM1_ESM.docx]

| **Additional File One: Example Template Summary Table** | |
| --- | --- |
| **CFIR Domain** | Participant number: 1 |
| **Innovation Characteristics** |  |
| 1. Innovation Source | *So I was involved pretty much from the outset of the scoping of the service so it was a recognition that patients were going into hospital either by ambulance whose needs could be better met in the community.* |
| 1. Evidence Strength & Quality | *So a little group was formed to identify what the opportunities were, and I worked with the others with the data and list and (Acute Hospital Trust) and we came up with a data set that we thought would be useful. So broadly speaking off the top of my head it was around patients were by ambulance to (Acute Hospital Trust), were discharged within 24 hours of arrival, whether for an ED or from, er, a decision making unit or indeed a ward with limited diagnostic codes, indicate.* |
| 1. Trialability | *I was involved in the initial pilot and the initial, er, evaluation, are we on the right track, you know, is this what I think it is, as opposed to this more formalised evaluation, what are we doing right, what are we doing wrong, what’s the lessons learned.* |
| **Outer Setting** |  |
| 1. Patient Needs & Resources of Those Served by the Organization | *There’s evidence in terms of patient’s physical and mental wellbeing. So physical in terms of the minute they go through that door we take away their, much of their mobility. Especially with this cohort of patients that we’re talking about they’ve probably already got some reduce mobility potentially anyway either mechanically or through illness. So, er, so if they’ve got COPD they probably might not be doing as much exercise as they previously did. And then we put them in a bed and they get even less exercise so their muscle tone, mechanical reasons why they’re not mobile, that’s exacerbated by them not having that mobility. Er, you’ve also got, then the physical side so nutrition, are they hydrated and nutritionally fed, in the same way that they were. Really simple – really simple things you know, how do they manage their continence, when you have to ask for help.*  *Which links to that mental health because you know take away someone’s, er, independence, you take away that independence will vary from individuals, it may just be watching the telly all day, or maybe you know out the front door standing and talking to neighbours and having visitors. All of those things are taken away. And obviously physical, as soon as you walk through a health centre you’re exposed to potential infection that you wouldn’t in your own community.* |
| 1. Networks and Communication | *I think there was a focus on CRIS, you can’t divorce CRIS from the central access point which is the unscheduled care co-ordination centre. And I think sometimes because you know CRIS are desperately trying to keep patients out of hospital, er, that there will be cases that come through to the unscheduled care co-ordination centre that don’t technically fit the criteria for CRIS, but they accept that to do the right thing and because there are or because there are internal blocks. Or other factors that have led to someone contacting unscheduled care co-ordination centres because they don’t contact CRIS, they contact the unscheduled care co-ordination centre. I think that’s a really important point in terms of this evaluation is the place that the UCCC holds in this chain*. |
| **Inner Setting** |  |
| 1. Culture   (norms and values of the CRIS) | *I think the challenge is around the access to other services … but they accept that to do the right thing.* |
| 1. Tension for Change | *All I’m trying to do is try and keep these patients out of hospital. So if through that gateway isn’t as broad as it could be, so then if I’m told, oh you need to contact, call community service, well we haven’t community services on the directory. Or I’m told I need to contact the palliative services or whatever it might be, well hang on you’re the unscheduled care co-ordination centre, why can’t you manage my need. Or the patient, more importantly the patient’s needs so yes there are capacity issues and this is why a lot of these patients are ringing 999 because their needs are, something has gone wrong elsewhere in the system, phone 999 or 111. Are the symptoms exaggerated or whatever or subjectively when they have got worse, and then you’ve got an issue alternative for this patient. And those alternatives can be thin on the ground so therefore CRIS can become a default to that. But the provider of CRIS, the provider of core community services and the provider of the unscheduled care co-*ordination centre are one and *the same.* |
| 1. Goals & Feedback | *I think cause and effect is really, really difficult. Especially it hasn’t helped the pandemic, it came at the time, I think you can say from certainly the first 12 months when there was lots of meetings about how’s it going, feedback. There was an incredible amount of qualitative feedback about great I was able to lead this patient to home and pass them on to, you know, to the service. There was lots of patient feedback as well, positive patient feedback that was reported in the meetings.* |
| 1. Access to Knowledge & Information | *So the event is a good way of doing it because you can pick topics. And I’ve just tied in the operational manager to reinstate those events.* |
| **Characteristics of Individuals** |  |
| 1. Knowledge & Beliefs about the Innovation | *I think other services did have this rapid response element things like that. But I think the aspiration of CRIS was, I’m not going to say unique, but was one that I wasn’t familiar with in terms of the extent that they wanted to address patient’s needs.* |
| 1. Individual Identification with Organization | *You know I’ll stay here for two years and then move to the next job, that’s the thing, if you’ve got a can culture that as far as I can see in my service is trying to establish.* |
| **Process** |  |
| 1. Planning | *This formalised pathway that’s been established gets complicated.* |
| 1. Engaging |  |
| 1. Opinion Leaders | *I think so, I think there has been, there was something of a sea change as well around getting people in the room to talk constructively about something. As opposed to, er, defensive conversations and, er, silo, the NHS has been brilliant over 35 years and we’re brilliant at silo working. I think what this did was recognise that CRIS wasn’t going to be successful on its own because it needed to get referrals so how was it going to get referrals, who was it going to get referrals from. And how those referrals remained and how would the right patients be identified. So there’s a whole reason for people being brought into a room to talk and talk in a constructive manner, which isn’t always the case within the NHS.* |
| 1. Formally Appointed Internal Implementation Leaders | *I’m going to say, you know, (leader of the service) in that regard, that inspirational leadership that’s been provided and prepared to, not run rough shod over things and tell people the way it should be. But what needs to be done to make things happen so, er, if we’d got more leaders like that, that’s what, also you know she will have the difficult conversations.* |
| 1. Innovation Participants | *Virtual wards.* *If they’re a means of ensuring that patient has access to the health care that they need, and the governance arrangements and risk management arrangements are consistent with what you’d expect for someone in a healthcare setting.* |
| *Domains with no corresponding data have been omitted | |
