# Supplementary material for: Applying the consolidated framework for implementation research to evaluate the community rapid intervention service
Source: BMC Health Serv Res. 2023 Aug 9;23:844. doi: 10.1186/s12913-023-09864-z (PMC10413526; doi:10.1186/s12913-023-09864-z)
Supplement: Supplementary file 2 — Additional file 2. Example from matrix. [file 12913_2023_9864_MOESM2_ESM.docx]

| ***Additional File Two: Example from matrix*** | | | | |
| --- | --- | --- | --- | --- |
| ***Theme*** | **CFIR domain** | **CFIR Construct** | **Description** | **Quotes/ Evidence** |
| Service identity | Intervention characteristics | Adaptability | The CRIS service adapted to meet the demands on the health services. The flexibility of the service was deemed both a strength and weakness. Participants suggested that the lack of clarity regarding inclusion/ exclusion criteria meant that the service could pick up patients that might ‘slip through the gaps’. However, the lack of clarity and inconsistency made the remit of the CRIS unclear. | *“Everybody – you know, the CRIS is the answer to everybody’s problems.” (Participant One)*  *“I think during COVID they then ended up getting involved in some other stuff with care homes which muddied the water even more. They then got involved with – I think they got involved with the long-winded COVID pathway, they got involved with – I can’t remember how many things that CRIS was the answer to during that time. So it’s lost. It seems very reactive though.” (Participant Nine)*  *“It makes sure patients don’t slip through the gaps” (Participant Seven)* |
|  |  | Complexity | The constructs adaptability and complexity were inter-linked. The adaptability of the service made the CRIS complex by nature as participants were unsure of service offering, or how to navigate the service. | *“What problem is the CRIS service trying to fix? And if the CRIS service is going to fix everybody’s problems then is just needs to stay as it is and just become a bigger and bigger monster.” (Participant Nine)*  *“This formalised pathway that’s been established gets complicated.” (Participant One)* |
